# Exploring Immune‐Related Gene and Mechanisms in Rosacea Through Transcriptome Analysis and Mendelian Randomization

**DOI:** 10.1155/bmri/7294117

**Published:** 2026-05-11

**Authors:** yafang wang, jian zhang

**Affiliations:** ^1^ The First People′s Hospital of Yinchuan, Yinchuan, Ningxia, China; ^2^ Ningxia Medical University, Second Clinical Medical College, Yinchuan, Ningxia, China, nxmu.edu.cn

**Keywords:** immunity, Mendelian randomization, rosacea, treatment

## Abstract

**Background:**

Dysregulation of the innate and adaptive immune system is thought to be central to the pathogenesis of rosacea. However, the molecular mechanism of immune‐related genes has not been extensively studied in rosacea.

**Methods:**

The GSE65914 and GSE155141 datasets were included in this study. Firstly, intersection genes were screened by overlapping key module genes obtained from weighted gene coexpression network analysis (WGCNA) and DEGs (rosacea vs. control). Then, functional enrichment analysis was performed to explore the functions of intersection genes. Subsequently, exposure factors (key genes) for rosacea were identified through Mendelian randomization (MR), and machine learning and expression analysis were conducted based on intersection genes. Single‐gene gene set enrichment analysis (GSEA) was utilized to investigate the molecular mechanisms of key genes. Additionally, a regulatory network was constructed. Finally, the mRNA‐drug interaction network was established.

**Results:**

A total of 624 intersection genes were screened, with functional enrichment results indicating involvement in positive regulation of protein kinase activity and the Hippo signaling pathway. Four key genes (ALDH1A1, COL17A1, RELL1, and ZNF404) were identified, with ALDH1A1 as a risk factor and the others as protective factors for rosacea. Single‐gene GSEA results showed enrichment in the IL‐17 and chemokine signaling pathways. The regulatory network included four mRNAs, two transcription factors (TFs), and 61 miRNAs. Lastly, 73 drugs targeting key genes were predicted.

**Conclusions:**

This study identified four immune‐related key genes (ALDH1A1, COL17A1, RELL1, and ZNF404) through MR analysis and machine learning, suggesting potential diagnostic or therapeutic candidates for further validation.

## 1. Introduction

Rosacea is a chronic inflammatory disease primarily affecting the central facial region, characterized by inflammatory reactions such as telangiectasia, skin thickening, and nodules [1]. The clinical features of rosacea include recurrent flushing, persistent erythema, inflammatory papules/pustules, and telangiectasia, which can progress to skin fibrosis in later stages. The prevalence of rosacea ranges from 1% to 22%. It is more common in women than in men, with a peak incidence between 45 and 60 years of age. The facial flushing and pustular‐like rash associated with this condition significantly impact the appearance of patients, leading to embarrassment, self‐confidence issues, anxiety, and potential depression. Consequently, it has a great impact on the psychological well‐being and quality of life of affected individuals. Contemporary therapeutic approaches prioritize the management of symptoms, mitigating the impact of the condition, and delaying or preventing disease progression. For individuals with mild presentations, topical antimicrobial, anti‐inflammatory, and keratolytic agents are utilized for localized treatment, including medications such as metronidazole and ivermectin. In cases of greater severity, systemic anti‐inflammatory agents may be required, such as tetracyclines, macrolides, or isotretinoin [2]. Additionally, laser and phototherapy are implemented for the treatment of telangiectasia, persistent erythema, and skin thickening associated with certain types of rosacea. Although current treatment strategies are diversified, limitations remain, particularly in the sustainability of remission and the rates of recurrence. Given these challenges, there is an imperative need to rigorously investigate existing therapeutic mechanisms and explore novel treatment pathways to furnish a more robust scientific foundation and a standardized therapeutic framework for the management of rosacea [3].

The formation and progression of rosacea are closely related to the regulation of the immune system. Specifically, it involves complex interactions between the innate and adaptive immune responses within the human body [3]. In patients with rosacea, studies have indicated that abnormal responses of immune cells may contribute to the exacerbation of inflammatory reactions. These responses are not limited to the superficial layers of the skin but can also extend to deeper cellular structures and tissues. Therefore, the imbalance and dysregulation of the immune system are focal points of research interest, which are not only pivotal in unveiling the underlying mechanisms of the disease but also essential in developing new therapeutic strategies targeting rosacea. The exact pathogenesis of rosacea remains unclear, although dysregulation of both the innate and adaptive immune systems is thought to play a central role. Studies have confirmed abnormal infiltration of polarized Th1/Th17 cells, macrophages, and mast cells in the perilesional areas and major subtypes of rosacea. Furthermore, early stages of rosacea show neutrophil infiltration, while B cells and plasma cells are involved in the late‐stage progression. Therefore, the identification and characterization of key immune genes in rosacea is crucial in unraveling its underlying mechanisms and providing important references for the clinical diagnosis and treatment of this condition [4].

To further investigate the causal relationship, strength of association, and directionality between key immune genes and rosacea, we employed Mendelian randomization (MR) in the second part of our study. MR is a specific type of instrumental variable (IV) analysis that utilizes genetic variation as IVs to detect and quantify causal relationships. In recent years, the application of MR in observational studies has become increasingly widespread due to its ability to overcome potential confounding variables and reverse causation. However, to date, there have been limited achievements in MR studies related to rosacea. For instance, Li et al. discovered a causal relationship between Crohn′s disease and rosacea through MR, with the variation in the interleukin‐23 receptor gene being a key factor influencing the development of rosacea. Therefore, MR can serve as an important tool in the study of the pathogenesis of rosacea.

In this study, based on transcriptomic data of rosacea patients from public databases, we screened for four immune key genes related to rosacea using a combination of biological methods and MR analysis. We determined the causal relationship between these genes and rosacea through MR. Additionally, we investigated the relevant pathways enriched by these key genes and explored therapeutic drugs targeting these genes, providing new clues for the study of rosacea treatment and offering potential avenues for further research in rosacea therapy [5].

## 2. Materials and Methods

### 2.1. Source of Data

The GSE65914 and GSE155141 datasets were sourced from the Gene Expression Omnibus (GEO) database (https://www.ncbi.nlm.nih.gov/geo/). The GSE65914 dataset (GPL570) included the microarray data of facial biopsy tissue from 20 control and 38 rosacea samples. The GSE155141 dataset (GPL10295) included the RNA‐seq of skin tissue from five rosacea patients and 10 control samples.

### 2.2. Acquisition of Differentially Expressed Genes (DEGs)

DEGs between the rosacea and control groups were selected in the GSE65914 dataset by using the limma package [6] (v 3.50.1) with adjusted *p* value < 0.05 and |log2*F* *C*| > 1. The |log2*F* *C*| > 1 threshold was chosen based on common practice in transcriptomic studies to identify genes with substantial expression changes, thereby reducing the likelihood of false positives and focusing on biologically meaningful differences. Following that, the volcano plot and heat map were drawn with the ggVolcano package (v 0.0.2) and Complex Heatmap package [7] (v 2.10.0), respectively, illustrating the variance of DEGs.

### 2.3. Key Module Genes Were Screened by Weighted Gene Coexpression Network Analysis (WGCNA)

The infiltration proportions of the 22 immune cells were computed using the CIBERSORT algorithm [8], and the Wilcoxon test was utilized to test for differences in immune cells between the rosacea and control groups. Differential immune cells were used as traits for further analysis. WGCNA was performed to construct coexpression networks [9] (v 1.71) to screen for genes associated with immune cells. Initially, clustering was performed on the samples to weed out outliers and ensure the accuracy of the analysis. Then, the optimal soft threshold (*β*) was selected to construct a scale‐free network. Immediately next, the clustering dendrogram was acquired by calculating the adjacency and similarity. The division of modules was carried out based on the dynamic tree‐cutting algorithm. Immediately following, we evaluated the correlation between each module and immune cells and selected the module with the highest correlation with the largest number of immune cells as the key module. Lastly, the genes in the key module were targeted as key module genes for the following analyses.

### 2.4. Acquisition and Functional Annotation of Intersection Genes

The given package (v 1.7.3) was utilized to take intersections of DEGs and key module genes to obtain intersection genes. Gene Ontology (GO) and Kyoto Encyclopedia of Genes and Genomes (KEGG) enrichment analyses of intersection genes were executed via the clusterProfiler package [10] (v 4.2.2) (adjusted *p* value < 0.05).

### 2.5. MR and Machine Learning Analysis

The intersection genes were used as exposure factors and rosacea as the outcome variable for MR analysis. Based on the selected exposure factors, LASSO analysis was performed using the glmnet package [11] (v 4.1‐2) to obtain candidate genes. We chose the LASSO approach over alternative feature selection methods (e.g., stepwise regression) because it is particularly robust in high‐dimensional settings and reduces model overfitting by applying an L1 penalty that shrinks the coefficients of noninformative variables to zero. Differences in the expression of candidate genes were compared in the GSE65914 and GSE155141 datasets, and genes with consistent expression trends were identified as key genes.

In order to clarify the causal association of key genes with rosacea, we carried out MR analyses. Key genes were used as exposure factors and rosacea as the outcome. The genome‐wide association study (GWAS) summary statistics for rosacea were obtained from https://www.ncbi.nlm.nih.gov/geo/. Firstly, the reading and filtering of exposure factors were carried out via the extract instruments function of the two‐sample MR package [12] (v 0.5.6) with *p* = 5 × 10^−8^, clump = TRUE, and *r* ^2^ = 0.001. The outcome variables were then read and filtered by the extract outcome data function of the two‐sample MR package [12] (v 0.5.6), and the effect alleles and effect sizes were harmonized by the harmonized function. MR analysis mainly consisted of five algorithms: MR‐Egger [13], weighted median, simple mode, weighted mode, and inverse variance weighted (IVW) regression [14]. The results mainly referred to the exposure factors obtained from the IVW algorithm. Then, odds ratios (ORs) were calculated, with values greater than 1 being a risk factor and less than 1 being a protective factor. The results were presented using scatter plots and funnel plots. The reliability of the results of the MR analyses was assessed through sensitivity analyses, which included the heterogeneity test, the horizontal pleiotropy test, and the leave‐one‐out (LOO) method. The final screened significant exposures were key genes for this study.

### 2.6. Single‐Gene GSEA

The single‐gene GSEA was performed to find the enriched regulatory pathways and biological functions of key genes with a *p* value < 0.05 and |*N* *E* *S*| > 1. The Top 5 results for KEGG were visualized separately.

### 2.7. Construction of a Regulatory Network

The transcription factors (TFs) and miRNA targeting the key genes were predicted using the miRNet database (https://www.mirnet.ca/). Then, the regulatory network was obtained by Cystoscope [15].

### 2.8. Construction of Key Gene–Drug Interaction Network

The drug with key gene interactions was predicted from the CTD database (https://ctdbase.org/). A key gene–drug network was constructed based on the predicted results. Then, the network was visualized using Cystoscope software [16].

### 2.9. Statistical Analysis and Quality Control for Transcriptomic Data

All bioinformatics analyses were carried out in R 4.5.1. The Wilcoxon test was utilized for comparison of differences between groups. MR analysis followed the MR‐STROBE guidelines, and data handling adhered to FAIR principles where applicable.

## 3. Results

### 3.1. Identification of DEGs and Key Module Genes

**Figure 1:**
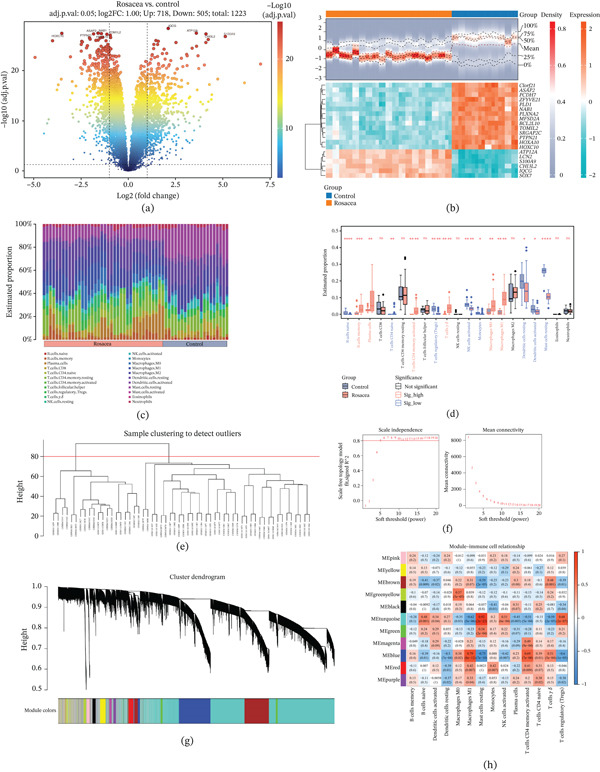
Identification of differentially expressed genes (DEGs) and key module genes related to immune cell infiltration. (a) Volcano plot of DEGs. (b) Heatmap of DEGs. (c) Analysis of the abundance of 22 immune cell infiltrations. (d) Differences in 22 types of immune cells. (e) Cluster analysis in weighted gene coexpression network analysis (WGCNA). (f) Optimal soft‐thresholding power selection for WGCNA. (g) Dendrogram based on a dissimilarity metric for differential immune cells. (h) Heatmap showing correlations between modules and immune cells. ^*^: Significant difference, ^**^: Very significant, ^***^: Highly significant, ^****^: Extremely significant, ns: No significant difference.

In total, 1223 DEGs between the rosacea and control groups were gained (718 upregulated and 505 downregulated) from the GSE65914 dataset (Figure 1). Among the Top 20 DEGs, there were 14 downregulated (*C1orf21*, *ASAP2*, *PCDH7*, *ZFYVE21*, *PLD1*, *NAB1*, *PLXNA2*, *MFSD2A*, *BCL2L10*, *TOM1L2*, *SRGAP2C*, *PTPN21*, *HOXA10*, and *HOXC10*) and six upregulated (*ATP12A*, *LCN2*, *S100A9*, *CHI3L2*, *IQCG*, and *SOX7*) in the rosacea group (Figure 1). Analysis of variance yielded 14 differential immune cells (activated dendritic cells, resting mast cells, and so on) between the rosacea and control groups (Figure 1). Among them, naive B cells, monocytes, and so on were less infiltrated in the rosacea group, while macrophages, plasma cells, and so on were more infiltrated in the rosacea group (Figure 1).

WGCNA was carried out in the GSE65914 dataset to find genes related to differential immune cells. The sample clustering results showed that there were no outlier samples (Figure 1). The interactions between genes maximally conformed to a scale‐free distribution when the soft threshold was equal to 10, *R* ^2^ = 0.85, and the average connectivity was close to zero (Figure 1). Then, a total of 11 modules were obtained (Figure 1). Of these, ME turquoise had a higher correlation with most immune cells (Figure 1). Hence, this module was regarded as a key module, and 7798 genes were recognized as key module genes.

### 3.2. Functional Enrichment of Intersection Genes

**Figure 2:**
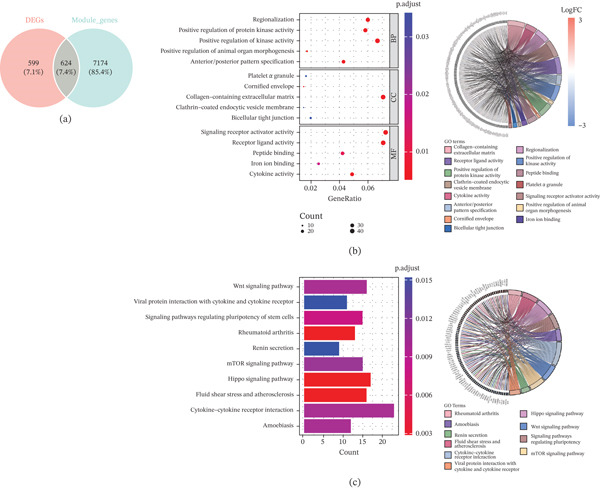
Identification of intersection genes. (a) Venn diagram of DEGs and key module genes. (b) GO analysis of intersection genes. (c) KEGG analysis of intersection genes.

In total, we obtained 624 intersection genes (*NAB1*, *HOXC10*, *HOXA10*, *CHI3L2*, *PTPN21*, *TOM1L2*, *S100A9*, etc.) by taking intersections of key module genes and DEGs (Figure 2). Next, the results of the enrichment analysis indicated that the intersection genes implicated 443 GO entries (CC: 10, MF: 20, and BP: 413) and 20 KEGG pathways. The intersection genes were mainly enriched in GO entries such as positive regulation of protein kinase activity, positive regulation of kinase activity, and so on (Figure 2). The Wnt signaling pathway, mTOR signaling pathway, Hippo signaling pathway, and so on were enriched in KEGG pathways (Figure 2).

### 3.3. Screening of Exposure Factors for Rosacea

**Figure 3:**
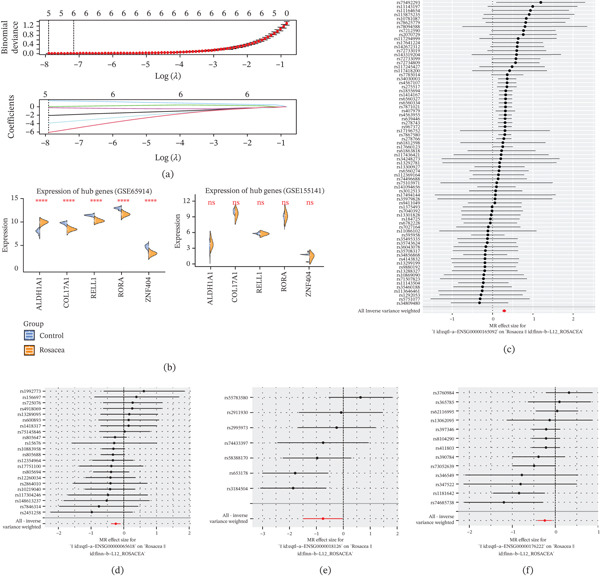
Screening of key genes. (a) Candidate genes were identified by LASSO logistic regression analysis. (b) The boxplot of candidate genes. Training set above and validation set below. The forest maps for (c) ALDH1A1, (d) COL17A1, (e) ZNF404, and (f) RELL1 with rosacea’s MR analysis.

Through MR analysis, we obtained a total of 11 genes that were causally associated with rosacea (Table 1). Subsequently, five candidate genes (*ALDH1A1*, *COL17A1*, *RELL1*, *RORA*, and *ZNF404*) were acquired by LASSO analysis (Figure 3). Expression analysis showed four genes (*ALDH1A1*, *COL17A1*, *RELL1*, and *ZNF404*) with consistent expression trends in the GSE65914 and GSE155141 datasets, with *ALDH1A1* highly expressed in rosacea, and *COL17A1*, *RELL1*, and *ZNF404* in contrast (Figure 3). Hence, these four genes were subsequently analyzed as key genes in this study.

**Table 1:** MR analysis of genes with a causal association with rosacea.

Symbol	ID.exposure	Outcome	pval
COL17A1	eqtl‐a‐ENSG00000065618	Rosacea || id:finn‐b‐L12_ROSACEA	0.033
RORA	eqtl‐a‐ENSG00000069667	Rosacea || id:finn‐b‐L12_ROSACEA	0.026
TP53INP2	eqtl‐a‐ENSG00000078804	Rosacea || id:finn‐b‐L12_ROSACEA	0.036
P3H2	eqtl‐a‐ENSG00000090530	Rosacea || id:finn‐b‐L12_ROSACEA	0.045
ZDHHC2	eqtl‐a‐ENSG00000104219	Rosacea || id:finn‐b‐L12_ROSACEA	0.012
SERPINE2	eqtl‐a‐ENSG00000135919	Rosacea || id:finn‐b‐L12_ROSACEA	0.002
ALDHIA1	eqtl‐a‐ENSG00000165092	Rosacea || id:finn‐b‐L12_ROSACEA	0
SMCO4	eqtl‐a‐ENSG00000166002	Rosacea || id:finn‐b‐L12_ROSACEA	0.025
CKB	eqtl‐a‐ENSG00000166165	Rosacea || id:finn‐b‐L12_ROSACEA	0.01
ZNF404	eqtl‐a‐ENSG00000176222	Rosacea || id:finn‐b‐L12_ROSACEA	0.033
RELL1	eqtl‐a‐ENSG00000181826	Rosacea || id:finn‐b‐L12_ROSACEA	0.046

*Note:* OR > 1, indicating a risk factor; OR < 1, indicating a protective factor. Method: inverse variance weighted (fixed effects).

A total of four significant exposure factors were acquired by MR analysis, which were *ALDH1A1*, *COL17A1*, *RELL1*, and *ZNF404*. The IVW method revealed a causal relationship between *ZNF404* (*p* = 0.0032), *ALDH1A1* (*p* = 3.37 × 10^−42^), *COL17A1* (*p* = 0.0008), and *RELL1* (*p* = 0.0428) with rosacea (Table 2). It was found that *ALDH1A1* (ORs = 1.3345) was a risk factor for rosacea, and *COL17A1* (OR = 0.7835), *ZNF404* (OR = 0.7769), and *RELL1* (OR = 0.4703) were protective factors for rosacea by observing the OR values (Figures 3, 3e, 3f, and, 3g and Table 2). The results of the five algorithms were plotted as scatter plots, and the results were consistent with the previous ones, with a small intercept and a positive (*ALDH1A1*) or negative (*COL17A1*, *ZNF404*, and *RELL1*) slope for IVW. The funnel plots showed that the sample distributions for the factor were left–right symmetrical, consistent with Mendel′s second law of random grouping (Figure S1).

**Table 2:** MR analysis results of key genes with rosacea (IVW method).

ID.exposure	NSNP	SE	pval	OR
ALDH1A1	76	0.021202497	3.37e − 42	1.334586
COL17A1	22	0.073392644	0.000888764	0.783547
RELL1	7	0.372448253	0.042854066	0.47036
ZNF404	13	0.085571777	0.003184637	0.77695

*Note:* OR > 1, indicating a risk factor; OR < 1, indicating a protective factor. Outcome: Rosacea || id:finn‐b‐L12_ROSACEA.

### 3.4. Analyses of Exposure Factors

**Figure 4:**
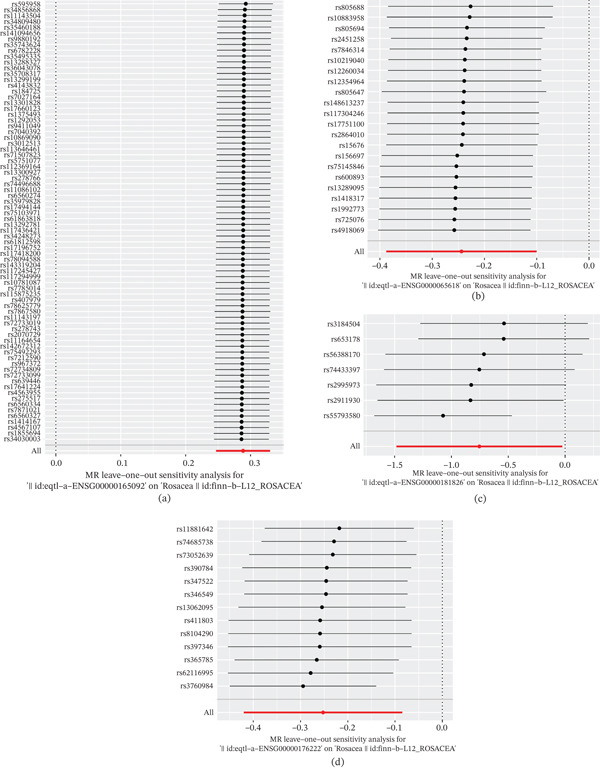
The results of the LOO analysis. The forest maps for LOO analysis in (a) ALDH1A1, (b) COL17A1, (c) ZNF404, and (d) RELL1 with rosacea.

The *Q*‐value of the heterogeneity test was less than 0.05, indicating that the sample was heterogeneous. There was no heterogeneity between the samples, with *Q*‐pvalue values greater than 0.05 (*ALDH1A1*: *Q* − pvalue = 0.939056924, *COL17A1*: *Q* − pvalue = 0.05148826, *RELL1*: *Q* − pvalue = 0.941952326, and *ZNF404*: *Q* − pvalue = 0.145443598) (Table 3). The results of the horizontal pleiotropy test indicated that there was no horizontal multieffect (*ALDH1A1*: *p* = 0.08716599, *COL17A1*: *p* = 0.187328512, *RELL1*: *p* = 0.169976874, and *ZNF404*: *p* = 0.863760101) (Table 4). In addition, the LOO analysis showed that the results were consistent with IVW and that the analysis was reliable (Figure 4, 4b, 4c, and, 4d). Hence, *ALDH1A1*, *COL17A1*, *RELL1*, and *ZNF404* were key genes for this study.

**Table 3:** Results of the heterogeneity test.

ID.exposure	*Q*	*Q*_df	*Q*_pval
ALDH1A1	57.0463486	75	0.939056924
COL17A1	11.9124083	21	0.941952326
RELL1	12.51125182	6	0.05148826
ZNF404	17.11170066	12	0.145443598

*Note:* Outcome: Rosacea || id:finn‐b‐L12_ROSACEA.

**Table 4:** Results of the horizontal pleiotropy test.

ID.exposure	Egger_intercept	SE	pval
ALDH1A1	−0.019980759	0.011526038	0.08716599
COL17A1	0.032636067	0.023904421	0.187328512
RELL1	−0.140452017	0.087653025	0.169976874
ZNF404	0.009373481	0.053364327	0.863760101

*Note:* Outcome: Rosacea || id:finn‐b‐L12_ROSACEA.

### 3.5. Single‐Gene GSEA of Key Genes

**Figure 5:**
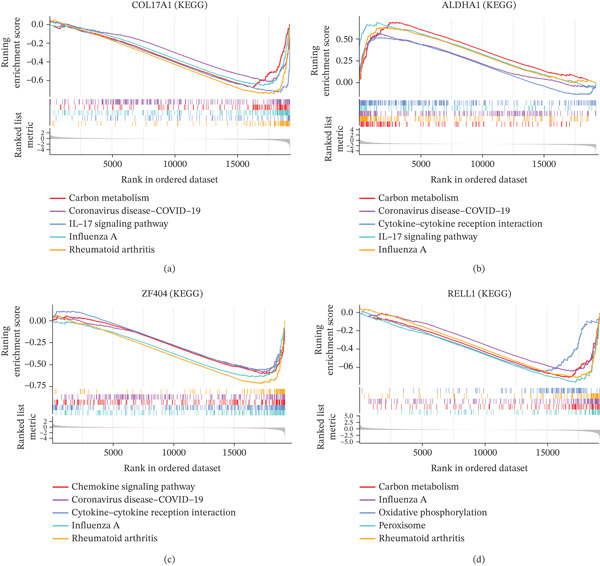
GSEA results. The function of (a) ALDH1A1, (b) COL17A1, (c) ZNF404, and (d) RELL1 using GSEA.

The functional enrichment results revealed that ALDH1A1 and COL17A1 were mainly enriched in KEGG pathways such as carbon metabolism, coronavirus disease (COVID‐19), and the IL‐17 signaling pathway (Figure 5). RELL1 and ZNF404 were mainly enriched in influenza A, rheumatoid arthritis, and so on (Figure 5). In conclusion, the functional enrichment results revealed that key genes were mainly enriched in KEGG pathways such as the IL‐17 signaling pathway, chemokine signaling pathway, and so on (Figures 5, 5b, 5c, and, 5d).

### 3.6. The Regulatory Networks of Key Genes

**Figure 6:**
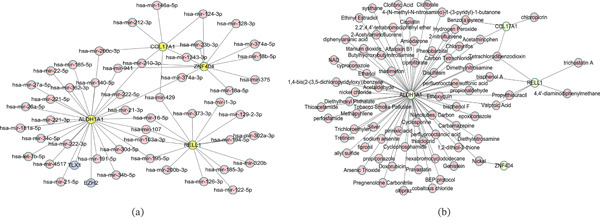
The regulated network of key genes. (a) The TF‐miRNA‐mRNA of ALDH1A1, COL17A1, ZNF404, and RELL1. (b) The drug networks of ALDH1A1, COL17A1, ZNF404, and RELL1.

To further investigate the regulatory mechanisms of key genes, we constructed a regulatory network. In total, two TFs and 61 miRNAs corresponding to key genes were identified. The network contained four key genes (ALDH1A1, COL17A1, RELL1, and ZNF404), 61 miRNAs (hsa‐mir‐375, hsa‐mir‐212‐3p, hsa‐mir‐103a‐3p, hsa‐mir‐27a‐5p, etc.), and two TFs (EZH2 and TLX1) (Figure 6). Among them, ALDH1A1 was targeted by all TFs and most miRNAs.

### 3.7. Prediction of Key Gene‐Related Drugs

We found four key genes targeted by 73 therapeutic drugs through the CTD database (Figure 6). The network included 70 drugs (acetaldehyde, acetaminophen, aflatoxin B1, etc.) for *ALDH1A1*, four drugs (benzo(a)pyrene, acetaminophen, chloropicrin, and tetrachlorodibenzodioxin) for *COL17A1*, six drugs (bisphenol A, propylthiouracil, tetrachlorodibenzodioxin, etc.) for *RELL1*, and one drug (nickel) for *ZNF404* (Figure 6).

## 4. Discussion

Rosacea is a chronic inflammatory skin disease primarily affecting the face, with its pathogenesis centered on dysregulation between the innate and adaptive immune systems. This study employed transcriptomic and metabolomic analyses to reveal key immune mechanisms in rosacea and identified four important immune‐related genes. Findings indicate that ALDH1A1 expression is upregulated in rosacea patients, while COL17A1, ZNF404, and RELL1 are significantly downregulated [17]. MR analysis confirmed that *ALDH1A1* was a risk factor [18], while *COL17A1*, *ZNF404*, and *RELL1* were protective factors. Network regulatory analysis and drug prediction highlighted the prominent role of *ALDH1A1* in the gene regulatory network. Network regulation analysis and drug prediction results indicated that ALDH1A1 plays a significant role in the gene regulatory network. Based on the above analysis, this study preliminarily revealed the potential roles of these four genes in rosacea, providing new directions for further understanding its molecular pathological mechanisms [1].

ALDH1A1 is an important enzyme for processing phthalates and plays a central role in maintaining metabolic stability [19]. The study found that ALDH1A1 significantly improved involvement in immune inflammation through the pathway to G‐17 transfusion and precipitation [20]. IL‐17 activates a known regulator of T‐cell divergence, contributes to the release of inflammatory cell factors, and alters the function of angle proteins [21]. This shows that ALDH1A1 can exacerbate WHAC diseases by enhancing Th17 inflammation. In contrast, a reduction in COL17A1 could hinder the repair of skin symptoms, while a reduction in RELL1 signaling could reduce disease control due to the suppression of NW‐B signaling [22]. Furthermore, ZNF404 exerts anti‐inflammatory properties by controlling the activity of immune cells. Together with the results, it is suggested that the anomaly in genetic regulation provides a molecular basis for further understanding of the mechanism by promoting the development and persistence of alcohol problems through immune regulators. This study not only aligns with previous research on the molecular mechanisms of rosacea but also expands current understanding in several critical aspects. Previous studies have confirmed that gene expression profiling and GWAS play a key role in elucidating genes associated with rosacea‐related immune inflammation and hyperpigmentation. Feng et al. noted that despite limited existing research, specific genetic variants may participate in immune regulatory pathways [23].

Recent evidence indicates that several DEGs are closely associated with the pathogenesis of rosacea [24]. For instance, the neutrophil activation marker S100A9 has been demonstrated to participate in the underlying inflammatory response of rosacea [25]. Furthermore, the Wnt, mTOR, and Hippo signaling pathways have been identified as key regulators of inflammatory amplification, vasodilation, and epidermal/vascular remodeling in rosacea [26]. Abnormal activation of these pathways promotes immune cell mobilization, angiogenesis, and keratinocyte dysfunction, thereby exacerbating lesion progression [27]. Our integrated analysis combined multiomics and genetic data. It revealed several immune‐related genes that influence key signaling pathways. These genes appear to form a bridge between molecular changes and clinical traits [28]. The connection reflects how cellular dysfunction gradually develops into tissue‐level abnormalities. This model offers a clear view of disease progression at different biological scales. MR analysis showed a causal link between these genes and rosacea. The results support a directional genetic effect. This method has also been used to study causal relationships between rosacea and environmental factors. Gut microbiota imbalance and smoking are two notable examples. Together, these findings enhance the credibility and clinical significance of causal reasoning in rosacea research [29]. Drug–gene interaction mapping indicated that retinoic acid and its analogs may be promising therapeutic candidates. They act on the ALDH1A1‐related pathway. Recent clinical studies have shown that low‐dose isotretinoin and similar retinoids are effective for papulopustular and treatment‐resistant rosacea [30]. Laboratory research also demonstrates that retinoic acid improves skin lesions. It promotes epithelial repair, reduces inflammation, and regulates sebaceous gland activity. These independent observations align with our pathway predictions [31]. They together provide a practical foundation for future studies targeting ALDH1A1 in rosacea treatment. Our work integrates molecular and genetic evidence. It connects immune‐related genes with well‐known signaling networks. This integration refines the current understanding of rosacea mechanisms [32]. It also provides a strong scientific rationale for developing targeted therapies focused on the ALDH1A1 axis. These hypotheses require further testing in large patient groups and experimental models [33], and future research will focus on elucidating the functional role of ALDH1A1 through gene knockout techniques in rosacea keratinocyte and vascular endothelial cell cultures, followed by validation in established animal models to assess its impact on disease‐related phenotypes.

Several limitations should be recognized. The sample size from public databases remains small. This limits how broadly the findings can be applied. Computational analysis also provides correlative rather than direct biological evidence. Experimental validation in cells and animal models is needed to confirm gene functions. MR has difficulty capturing interactions between genes and the environment. Such interactions may play major roles in rosacea. The study also focused on common variants, leaving rare mutations and epigenetic factors underexplored [34]. Future research should examine these gene–pathway links in larger and multicenter cohorts. Long‐term studies can track molecular changes during disease development and treatment response. Functional experiments using cellular and animal systems will clarify the roles of ALDH1A1, COL17A1, ZNF404, and RELL1. These genes may influence immune regulation, inflammation, and skin barrier recovery [35]. Integrating genomic, proteomic, and metabolomic information could help build a complete “gene–immune–metabolic” network model. Such a model would describe rosacea at a systems level. In clinical translation, evaluating the effects of retinoic acid derivatives on different rosacea types remains important. Combination therapies using immune regulators, antioxidants, or signaling inhibitors deserve exploration. The future direction involves combining artificial intelligence with multiomics integration. This approach may support molecular subtyping and personalized treatment prediction. It can help close the gap between biological discovery and clinical application. Overall, the study deepens understanding of rosacea′s molecular mechanisms. It offers a theoretical basis for developing precision therapies that target specific molecular pathways.

## 5. Conclusion

This study demonstrates that ALDH1A1 and related genes play a pivotal role in the pathogenesis of rosacea by regulating immune‐inflammatory pathways, providing a theoretical basis for precision therapy targeting ALDH1A1. However, larger sample sizes and experimental validation are still required.

## Author Contributions

yafang wang performed the research and analyzed the data. jian zhang designed the research study and wrote the paper. yafang wang and jian zhang contributed equally to this research.

## Funding

No funding was received for this manuscript.

## Disclosure

All authors read and approved the final manuscript.

## Conflicts of Interest

The authors declare no conflicts of interest.

## Supporting information


**Supporting Information** Additional supporting information can be found online in the Supporting Information section. The scatter plot and funnel plot of the MR analysis. The scatter plots for (A) ALDH1A1, (B) COL17A1, (C) ZNF404, and (D) RELL1 with rosacea′s MR analysis. The funnel plot for (E) ALDH1A1, (F) COL17A1, (G) ZNF404, and (H) RELL1 with rosacea′s MR analysis.

## Data Availability

The datasets generated and analyzed during the current study are available from the corresponding author upon reasonable request.
