# Supplementary material for: Exploring Immune‐Related Gene and Mechanisms in Rosacea Through Transcriptome Analysis and Mendelian Randomization
Source: Biomed Res Int. 2026 May 11;2026:7294117. doi: 10.1155/bmri/7294117 (PMC13159088; doi:10.1155/bmri/7294117)

Supplementary Figure 1The scatter plot and funnel plot of MR analysis. A-D The catter plots for ALDH1A1 (A), COL17A1(B), ZNF404 (C), and RELL1 (D) with rosacea's MR analysis. E-H The funnel plot for ALDH1A1 (E), COL17A1(F), ZNF404 (G), and RELL1 (H) with rosacea's MR analysis.


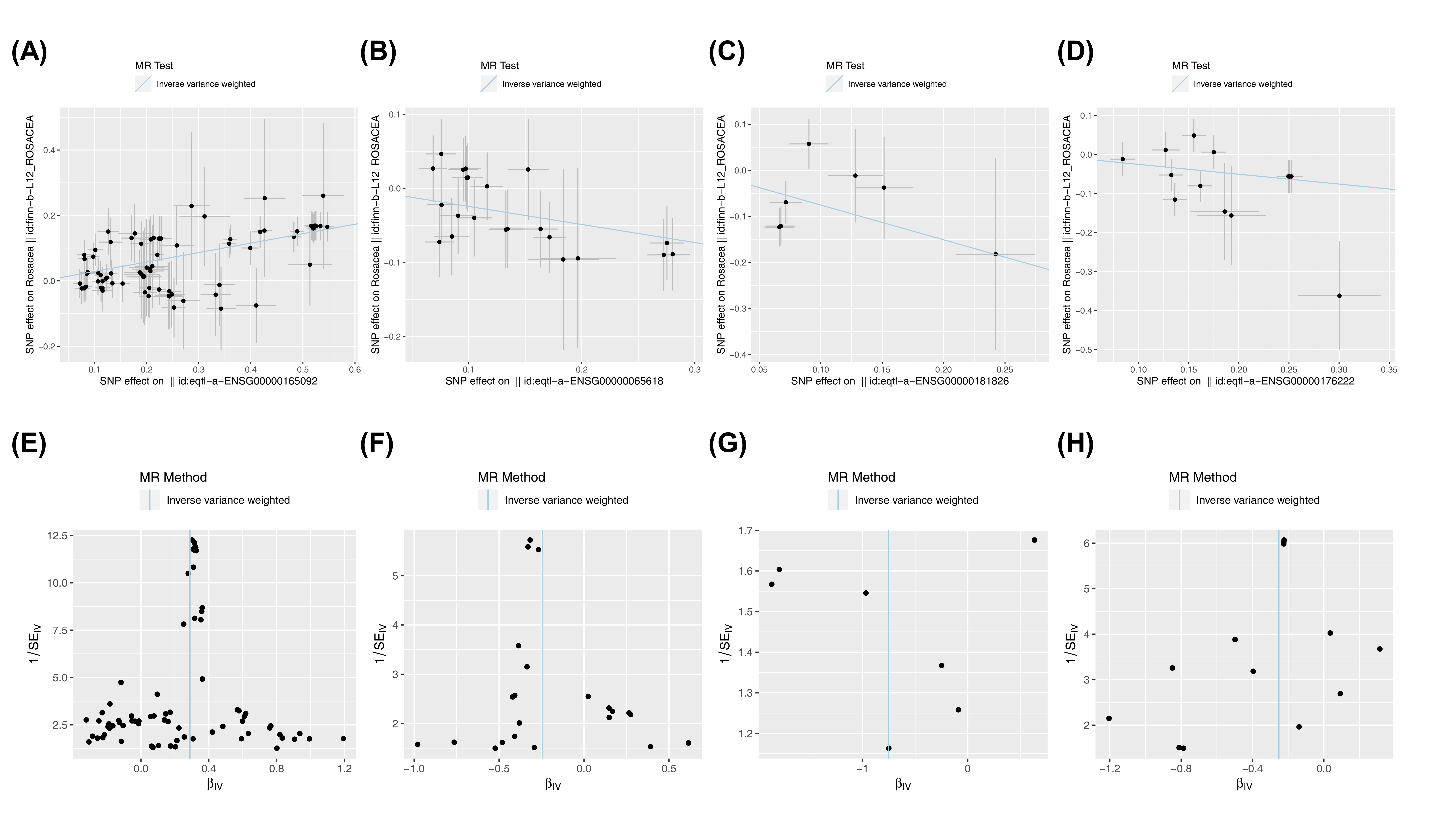

Supplement: Supplementary file 1 — Supporting Information Additional supporting information can be found online in the Supporting Information section. The scatter plot and funnel plot of the MR analysis. The scatter plots for (A) ALDH1A1, (B) COL17A1, (C) ZNF404, and (D) RELL1 with rosacea′s MR analysis. The funnel plot for (E) ALDH1A1, (F) COL17A1, (G) ZNF404, and (H) RELL1 with rosacea′s MR analysis. [file BMRI-2026-7294117-s001.docx]
